# Supplementary material for: Multiplex recombinase polymerase amplification assay developed using unique genomic regions for rapid on-site detection of genus Clavibacter and C. nebraskensis
Source: Sci Rep. 2021 Jun 8;11:12017. doi: 10.1038/s41598-021-91336-7 (PMC8187419; doi:10.1038/s41598-021-91336-7)
Supplement: Supplementary file 1 — Supplementary Information. [file 41598_2021_91336_MOESM1_ESM.pdf]

Multiplex recombinase polymerase amplification assay developed using unique genomic regions for rapid on-site detection of genus *Clavibacter* and *C. nebraskensis*

Adriana Larrea-Sarmiento[1], James P. Stack[2], Anne M. Alvarez[1], and Mohammad Arif[1*]

[1]Department of Plant and Environmental Protection Sciences, University of Hawaii at Manoa, Honolulu, Hawaii - 96822, United States of America

[2]Department of Plant Pathology, Kansas State University, Manhattan, Kansas – 66506, United States of America

*Corresponding author- Email: arif@hawaii.edu; Phone: +1-808-956-7765

SUPPLEMENTARY INFORMATION

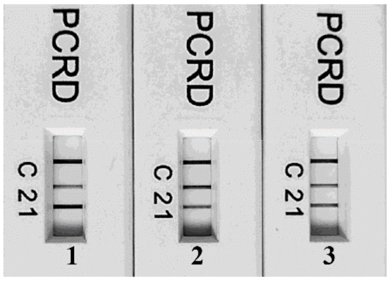

Fig. S1. RPA assay optimization for primer and probe concentration. Multiplex RPA reactions were performed following the provider instructions. *Clavibacter*-specific primer/probe concentrations were evaluated against *Cn*-specific primer/probe set. 1) low (*Cn* 1.2 µL/1.4 µL; *Clavibacter* 0.8 µL /0.6 µL); 2) equal (*Cn* 1.2 µL/0.8 µL; *Clavibacter* 1.2 µL/0.8 µL); and 3) high (*Cn* 1.5 µL/0.9 µL; *Clavibacter* 1.0 µL/0.6 µL).
